# Effect of Acetazolamide on Intraocular Pressure After Uneventful Phacoemulsification Using an Anterior Chamber Maintainer

**DOI:** 10.3390/vision9030073

**Published:** 2025-08-28

**Authors:** Assaf Kratz, Tom Kornhauser, Eyal Walter, Ran Abuhasira, Ivan Goldberg, Aviel Hadad

**Affiliations:** 1 Department of Ophthalmology, Soroka University Medical Center, Ben Gurion University of the Negev, P.O. Box 151, Beer Sheva 84101, Israel; 2 Department of Ophthalmology, Shamir Medical Center (Assaf Harofeh), Be′er Ya′akov 70300, Israel; 3 Clinical Research Center, Soroka University Medical Center, Faculty of Health Sciences, Ben Gurion University of the Negev, P.O. Box 151, Beer Sheva 84101, Israel; 4 Glaucoma Unit, Sydney Eye Hospital, GPO Box 1614, Sydney, NSW 2001, Australia; 5 Discipline of Ophthalmology, University of Sydney, Sydney, NSW 2006, Australia; 6 Eye Associates, Sydney, NSW 2000, Australia

**Keywords:** cataract, phacoemulsification, intraocular pressure spike, anterior chamber maintainer, acetazolamide, intraocular pressure spike prevention, acetazolamide, anterior chamber maintainer, cataract surgery, headache

## Abstract

**Background:** Transient intraocular pressure (IOP) elevations frequently occur after cataract surgery and may raise concerns, especially in patients susceptible to glaucomatous damage or pressure-related complications. These IOP spikes have also been linked to postoperative discomfort and headache. Oral acetazolamide is often used prophylactically, despite its known systemic side effects. **Objectives:** To evaluate the clinical benefit of routine prophylactic oral acetazolamide in reducing IOP after uncomplicated phacoemulsification performed with an anterior chamber maintainer (ACM). **Methods:** In this retrospective case–control study, 196 eyes from 196 patients were included. All underwent standard phacoemulsification with an ACM. Patients either received oral acetazolamide postoperatively (*n* = 98) or no IOP-lowering medication (n = 98). IOP was measured preoperatively, and on postoperative days one and seven. **Results:** On day one, mean IOP was 14.0 ± 3.8 mmHg in the acetazolamide group versus 15.4 ± 3.8 mmHg in controls (*p* < 0.005). By day seven, IOP was identical in both groups (13.5 mmHg), with no statistically significant difference (*p* = 0.95). No participant in either group reported headache or serious adverse effects, though 10% in the acetazolamide group experienced mild, transient systemic symptoms. **Conclusions:** In low-risk patients undergoing uneventful cataract surgery with ACM, routine use of oral acetazolamide yields only a modest, short-lived IOP reduction without evident clinical benefit. Its use may be unnecessary in this setting, though targeted prophylaxis could be considered for high-risk individuals.

## 1. Introduction

Transient increases in intraocular pressure (IOP) are a well-recognized phenomenon following cataract surgery. These postoperative IOP elevations are of particular concern in the current era of outpatient surgery, where early detection and intervention may be limited. While generally self-limiting, such IOP spikes have been associated with a range of adverse outcomes, including visual field deterioration in glaucomatous eyes, anterior ischemic optic neuropathy, retinal vascular occlusion and corneal edema [1,2,3,4,5,6,7,8]. Moreover, symptoms of headache occurred in 8% of cases, mostly in association with raised IOP [8].

Multiple mechanisms have been implicated in these pressure elevations, including residual ophthalmic viscosurgical devices (OVDs) obstructing the trabecular meshwork, lenticular debris, post-surgical inflammation, and variations in surgeon technique and experience [1,2,3,4,5,6,7,8]. Consequently, several pharmacologic strategies have been investigated to attenuate postoperative IOP spikes. These include intracameral miotics and a range of topical and systemic ocular hypotensive agents—among them timolol, travoprost, latanoprost, brimonidine, apraclonidine, dorzolamide, brinzolamide, and acetazolamide [9,10,11,12,13,14,15,16,17,18,19,20,21,22,23,24,25].

Modern cataract surgery, typically performed via phacoemulsification, relies on maintaining anterior chamber stability through continuous irrigation with balanced salt solution. Upon withdrawal of the phaco probe, the anterior chamber is often sustained using viscoelastic agents. To further stabilize the chamber and prevent sudden collapse, many surgeons employ an anterior chamber maintainer (ACM), which delivers a constant fluid inflow and provides enhanced intraoperative IOP stability [26,27,28,29]. At our institution, the use of ACMs is standard practice in routine cataract surgery.

As mentioned, many surgeons routinely prescribe oral acetazolamide postoperatively, a drug known to cause systemic adverse effects such as paresthesia, fatigue, gastrointestinal upset, and electrolyte imbalance. We hypothesized that the use of ACMs, by facilitating complete removal of OVDs and debris, might eliminate the need for such systemic prophylaxis.

The objective of this study was to determine whether the addition of prophylactic oral acetazolamide following uncomplicated phacoemulsification with an ACM yields a meaningful advantage in IOP control and prevention of early postoperative spikes.

## 2. Patients and Methods

This was a retrospective case–control study based on anonymized clinical records of patients who underwent uncomplicated phacoemulsification cataract surgery. The study was approved by the institutional review board of Soroka University Medical Center (authorization number 0058-17) and conducted in accordance with the tenets of the Declaration of Helsinki.

### 2.1. Patient Selection and Study Design

Patients were selected from two distinct time periods to reflect differing institutional practices regarding postoperative acetazolamide prophylaxis. The acetazolamide group comprised patients operated during a period in which routine administration of oral acetazolamide was standard institutional practice. The control group included patients from a subsequent period during which this practice was no longer routinely employed. All other aspects of the surgical technique and perioperative care, including the choice and formulation of OVDs, phacoemulsification fluidics parameters, and the postoperative anti-inflammatory regimen, remained identical between the two study periods.

Inclusion was limited to patients who had undergone uneventful phacoemulsification. Exclusion criteria included any form of glaucoma or ocular hypertension (OHT), pseudoexfoliation syndrome (PXF), prior intraocular surgery or trauma in the operated eye, known allergy to carbonic anhydrase inhibitors, renal failure, and any significant corneal opacity that could interfere with intraocular pressure (IOP) measurement. Cases involving any intraoperative complications, such as posterior capsular rupture, vitreous loss, or intraocular lens (IOL) implantation outside the capsular bag, were excluded as well.

### 2.2. Treatment Protocol and Measurements

Eyes were assigned to one of two treatment arms: The acetazolamide group received a regimen of 250 mg oral acetazolamide administered 15 min postoperatively, followed by two additional doses—one in the evening of the same day and the last dose on the morning of the following day. The control group received no prophylactic IOP-lowering medication.

A comprehensive ophthalmic examination was performed preoperatively and repeated on postoperative day one and day seven. IOP was measured at all time points using a Goldmann applanation tonometer (AT900, Haag-Streit, Köniz, Switzerland) by trained personnel. Best-corrected visual acuity (BCVA) was assessed preoperatively and again at the postoperative day seven visit.

### 2.3. Surgical Technique

All surgeries were performed by a single experienced surgeon, employing a standardized phacoemulsification technique. Procedures were conducted under topical and subconjunctival anesthesia using an Infiniti unit (Alcon, Inc. Fort Worth, TX, USA) from a 12 o’clock surgical approach.

A Blumenthal anterior chamber maintainer (20-gauge) delivering balanced salt solution (BSS) was inserted through a temporal paracentesis to maintain stable anterior chamber pressure throughout the procedure. A cohesive ophthalmic viscosurgical device (OVD), specifically sodium hyaluronate 2% (Visiol, TRB Chemedica International SA, Carouge GE, Switzerland), was injected into the anterior chamber prior to continuous curvilinear capsulorhexis and additionally as needed during surgery.

Following lens extraction, an acrylic foldable intraocular lens (Lentis L-312, Oculentis, GmbH, Berlin, Germany) was implanted into the capsular bag via an injector cartridge. No additional OVD was used during or after IOL insertion. Residual OVD and lenticular debris were removed using a manual syringe and cannula. Miotic agents were not administered.

At the end of surgery, unless contraindicated by penicillin allergy, 1 mg of intracameral cefuroxime in 0.1 mL was injected for infection prophylaxis. All surgical incisions were hydrated with BSS and checked for watertight closure. Intraocular pressure was assessed at the end of surgery by gentle indentation using a surgical cannula and gentle decompression was carried out when necessary to restore physiologic pressure.

### 2.4. Postoperative Treatment

Postoperative treatment included topical third-generation fluoroquinolone antibiotic drops (ofloxacin) and topical dexamethasone steroid drops. For the first three postoperative days, both medications were administered eight times daily, followed by four times daily until the one-week postoperative visit. Thereafter, treatment was continued or tapered according to the surgeon’s discretion.

### 2.5. Statistical Analysis

Power and sample size calculations were based on epidemiologic studies, where mean IOP in a population similar to our study population was found to be approximately 15 mmHg with a standard deviation of approximately 4 mmHg [30]. The power was set to 0.80 and alpha to 0.05. Significant difference for the tests was estimated to be 1.6 mmHg. For this difference, the sample size was calculated to be 196 eyes, 98 in each group.

Results are presented by mean ± standard deviation for continuous variables and as percentages for categorical data. Chi-square was used for categorical variables and *t*-test for continuous variables, including IOP.

To adjust for treatment indication bias, we used propensity score matching [31], which incorporates data from the independent variables into a logistic regression model predicting the probabilities of being assigned to acetazolamide prophylaxis prior to the cataract surgery. The outcome of the score was the postoperative IOP on days one and seven. The variables used to create the score were age, sex and the preoperative IOP. We then performed number matching with a caliper of 0.1.

Linear mixed models were used to estimate the association between the acetazolamide prophylaxis group and the postoperative IOP. Mixed models were used to account for the clustering of different eyes of the same patients and for repeated measurements of intraocular pressure using Autoregressive Moving Average (ARMA) repeated covariance type. Age, sex and preoperative IOP were defined as fixed effects.

SPSS IBM software, version 27.0, and Stata software, version 15.0, were used for statistical analysis.

## 3. Results

Following application of exclusion criteria and propensity score matching, a total of 196 eyes from 196 patients were included in the final analysis. All patients underwent uneventful phacoemulsification cataract surgery performed by the same surgeon, using a standardized technique. The mean age of the study population was 70.8 ± 10.4 years, and 77 patients (52.4%) were female.

The cohort was evenly divided between the acetazolamide group (n = 98 eyes) and the control group (n = 98 eyes). Baseline characteristics—including age and preoperative intraocular pressure—were statistically similar between the two groups (Table 1). Preoperative IOP was 15.2 ± 3.3 mmHg in the control group and 15.5 ± 2.9 mmHg in the acetazolamide group (*p* = 0.409).

**Figure 1:**
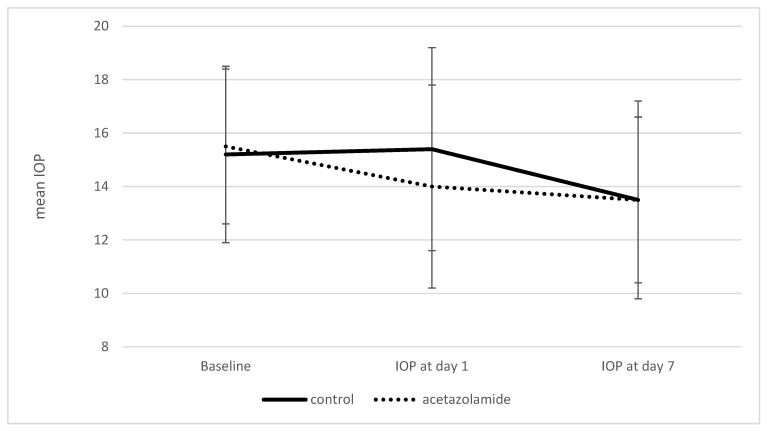
Mean IOP at baseline, postoperative day 1, and day 7 in the acetazolamide and control groups. Error bars represent 95% confidence intervals. IOP—intraocular pressure; Bars represents standard deviation.

On postoperative day one, the mean IOP was 15.4 ± 3.8 mmHg in the control group versus 14.0 ± 3.8 mmHg in the acetazolamide group, demonstrating a statistically significant difference in favor of the treatment group (*p* = 0.01). By postoperative day seven, mean IOP levels had decreased in both groups and were identical at 13.5 mmH ± 3.7 mmHg in the control group vs. 13.5 ± 3.1 mmHg in the acetazolamide group, with no statistically significant difference (*p* = 0.95), (Figure 1). A statistically significant reduction in IOP was observed in both groups by postoperative day seven when compared to preoperative measurements (*p* < 0.001 for both) (Table 2).

Across the entire study population, regardless of treatment group, preoperative intraocular pressure and age were significantly associated with postoperative IOP. Specifically, for each 1 mmHg increase in baseline IOP, postoperative IOP increased by 0.57 mmHg (95% CI, 0.46 to 0.69). Patients under the age of 65 had 1.6 mmHg higher postoperative IOP compared to those aged 75 and older (95% CI, 0.7 to 2.49). Sex was not significantly associated with postoperative IOP after adjustment for age and preoperative pressure (Table 3).

In the context of BCVA on postoperative day 7, several statistically significant associations emerged following propensity score adjustment. Assignment to the acetazolamide prophylaxis group was associated with a 0.07-unit decrease in mean LogMAR score, indicating slightly better visual acuity. Worse preoperative vision predicted poorer outcomes: each 1-unit increase in baseline LogMAR corresponded to a 0.18-unit increase postoperatively. Additionally, each additional year of age was linked to a 0.01-unit increase in postoperative LogMAR, and male sex was associated with a 0.05-unit higher score compared to females (Table 4).

Approximately 10% of patients in the acetazolamide group reported transient systemic side effects, including generalized weakness, decreased appetite, peripheral paresthesia, and altered taste perception (dysgeusia). These symptoms were mild, self-limited, and resolved completely by the first postoperative day. No serious or persistent adverse events were recorded in either group.

## 4. Discussion

Elevation of IOP in the early postoperative period following cataract surgery is a well-established concern, particularly in eyes with glaucoma or other predispositions to optic nerve damage. Transient IOP spikes may result from obstruction of the trabecular meshwork by OVDs, inflammatory debris, or lenticular fragments [1,2,3,4,5,6,7,8].

These pressure elevations, although often self-limiting, have prompted many surgeons to routinely administer prophylactic IOP-lowering agents [9,10,11,12,13,14,15,16,17]. Among these, oral acetazolamide remains a widely used option, owing to its systemic potency and rapid onset of action [18,19,20,21,22,23,24,25]. A UK-wide survey reported that 87% of ophthalmic consultants who routinely prescribe prophylaxis prefer oral acetazolamide over topical agents [21].

Prophylaxis of IOP spikes after cataract surgery relies on both surgical technique and pharmacologic intervention, particularly in high-risk patients such as those with glaucoma or PXF. The American Academy of Ophthalmology (AAO) emphasizes the importance of thorough removal of OVDs at the end of surgery, as retained viscoelastics—especially dispersive types—are strongly associated with postoperative IOP elevation. When dispersive agents are used, extra attention to irrigation and aspiration is warranted to minimize the risk of pressure spikes [5].

Pharmacologic strategies also play a central role in prophylaxis. Topical aqueous suppressants have demonstrated efficacy in reducing both the incidence and magnitude of IOP elevations, particularly when administered immediately after surgery [9,10,11,12,13,14,15,16,17]. Oral acetazolamide, administered pre- or postoperatively at a dose of 500 mg, has also shown effectiveness in reducing postoperative IOP elevation. These strategies are typically tailored based on patient-specific risk factors and tolerability profiles [18,19,20,21,22,23,24,25].

In our study, we assessed the clinical benefit of oral acetazolamide in patients undergoing uneventful phacoemulsification cataract surgery with the use of an anterior chamber maintainer (ACM). A statistically significant reduction of 1.4 mmHg in IOP was observed on postoperative day one in the acetazolamide group compared to controls. However, this difference was not sustained, and by postoperative day seven, mean IOP levels were identical in both groups (Figure 1). Notably, IOP values remained within normal physiological range throughout the follow-up period in both arms (Table 2). Therefore, while the short-term effect of acetazolamide was statistically significant, it is unlikely to be clinically meaningful in routine, low-risk cases.

Although topical corticosteroids can induce intraocular pressure elevation, both study groups received an identical postoperative regimen, including dexamethasone drops. Therefore, any potential steroid-related effect on intraocular pressure would have been equally distributed between the groups and thus unlikely to bias the results.

Our findings align with previous studies demonstrating the inherent IOP-lowering effect of cataract surgery itself, as both groups in our cohort exhibited a statistically significant reduction in IOP by postoperative day seven compared to baseline, regardless of whether acetazolamide was administered. Notably, this effect was observed in patients without glaucoma, consistent with the non-glaucomatous population included in our study [32,33,34].

In our cohort, each 1 mmHg increase in baseline intraocular pressure (IOP) was associated with a 0.57 mmHg increase in postoperative IOP. Although this association was statistically significant, its clinical relevance appears limited, as postoperative IOP values remained within normal physiological ranges. Additionally, patients under the age of 65 exhibited higher postoperative IOP compared to those aged 75 and above (Table 3). This age-related trend aligns with previous reports suggesting that older age is associated with a more pronounced IOP reduction following cataract surgery [1,33].

In our cohort, several statistically significant associations were identified between baseline characteristics and visual acuity on postoperative day 7 (Table 4). Specifically, acetazolamide use, poorer preoperative vision, older age, and male sex were each associated with slightly worse LogMAR outcomes; however, the magnitude of these differences was small and may not reflect clinically meaningful effects. Although the acetazolamide group demonstrated a statistically significant improvement in BCVA on postoperative day 7 compared to controls, the magnitude of this difference, approximately 0.07 logMAR (around three letters on a Snellen chart), is not clinically meaningful. In the absence of a plausible biological mechanism linking short-term acetazolamide use to such an effect, and considering the similar baseline characteristics between the groups, this difference is most likely the result of a statistical artifact (e.g., Type I error) rather than a true therapeutic benefit.

A unique aspect of our study is the systematic use of the anterior chamber maintainer (ACM) during surgery. This instrument helps preserve stable anterior chamber depth and facilitates precise intraoperative control by delivering continuous irrigation [26,27,28,29]. We postulated that, beyond its mechanical stabilization, the ACM may actively aid in clearing residual ophthalmic viscosurgical device (OVD) material and inflammatory debris, thereby reducing the risk of postoperative IOP elevation. The continuous “flushing” mechanism of the ACM throughout the procedure may have contributed to the stable and well-controlled IOP observed in the control group, despite the absence of pharmacologic prophylaxis. To our knowledge, no prior study has directly examined the combined effect of acetazolamide prophylaxis and ACM use on IOP outcomes.

Our findings are consistent with prior studies evaluating carbonic anhydrase inhibitors, which have shown postoperative IOP reductions in both glaucomatous and non-glaucomatous eyes [18,19,20,21,22,23,24,25]. The absence of clinically significant elevations and the parallel decrease in IOP observed in both groups by day seven support the notion that acetazolamide may not be necessary in routine cases when ACM is used effectively.

It is important to consider the side effect profile of systemic acetazolamide. In our study, approximately 10% of patients reported transient symptoms such as weakness, appetite loss, paresthesia, and dysgeusia, all self-limiting, but potentially bothersome. Avoiding unnecessary medication in low-risk patients may thus improve postoperative comfort and reduce systemic exposure.

Although headache was not specifically measured or recorded as an endpoint in our study, it was not reported spontaneously by any participant in either group. The absence of headache reports may suggest that neither the drug nor postoperative IOP changes caused clinically meaningful headache symptoms in this setting. This observation may further support the limited clinical value of routine prophylactic acetazolamide in uncomplicated phacoemulsification procedures performed with an anterior chamber maintainer.

This study has several limitations. As a retrospective analysis, it may be subject to selection bias; however, propensity score matching was employed to reduce this risk. In addition, IOP was not measured at frequent intervals during the immediate postoperative hours. While this might be a concern in detecting transient IOP spikes, the low IOP values recorded on postoperative day one in both groups suggest that clinically significant elevations were less likely. Therefore, repeated nocturnal IOP monitoring was deemed unnecessary in this specific clinical context.

A potential source of bias in this study is the fact that the two groups were drawn from different time periods, which might theoretically reflect subtle changes in surgical technique or perioperative care. However, in practice, all relevant surgical and perioperative parameters, including the type of OVD, phacoemulsification fluidics settings, postoperative anti-inflammatory regimen, and the operating surgeon, remained identical between the two study periods. The only variable that differed between the groups was the administration of acetazolamide.

It is important to note that the study was intentionally limited to non-glaucomatous patients undergoing uneventful phacoemulsification with the use of an ACM. This defined and homogeneous population allowed focused evaluation of acetazolamide prophylaxis in routine, low-risk cataract surgery. As such, the findings may not be generalizable to eyes with glaucoma, pseudoexfoliation, or other complicating factors. Finally, the follow-up period was limited to seven days, which precluded assessment of long-term IOP trends or delayed complications.

In conclusion, while oral acetazolamide remains a valid option for managing IOP in high-risk eyes, our data indicate that its routine prophylactic use following uneventful cataract surgery with ACM assistance offers limited added value and may be safely avoided in low-risk patients.

## Figures and Tables

**Table 1:** Baseline Demographic and Clinical Characteristics of the Study Population.

	Control (No. of Eyes = 98)	Acetazolamide (No. of Eyes = 98)	*p*-Value
Age (Mean ± SD)	70.3 ± 10.1	71.2 ± 10.6	0.578
Male	51 (52.0%)	38 (38.8%)	0.06
Female	47 (48.0%)	60 (61.2%)	
Mean preoperative IOP (mmHg, Mean ± SD)	15.2 ± 3.3	15.5 ± 2.9	0.409

IOP—intraocular pressure, SD—standard deviation.

**Table 2:** IOP values at postoperative days 1 and 7.

	Control (No. of Eyes = 98, Mean ± SD, mmHg)	Acetazolamide (No. of Eyes = 98, Mean ± SD, mmHg)	*p*-Value
Preoperative IOP	15.2 ± 3.3	15.5 ± 2.9	0.409
IOP at day 1	15.4 ± 3.8	14.0 ± 3.8	0.01
IOP at day 7	13.5 ± 3.7	13.5 ± 3.1	0.95

IOP—intraocular pressure; SD—standard deviation.

**Table 3:** Estimated effect of acetazolamide prophylaxis on intraocular pressure one and seven days after cataract surgery.

Variable	Effect Estimate (95% CI)	*p*-Value
Pre-operative IOP (mmHg)	0.57 (0.46 to 0.69)	<0.001
Age		
Under 65	1.6 (0.7 to 2.49)	0.001
65–74	0.78 (−0.04 to 1.6)	0.061
75 and older	0 (reference)	
Female	0.24 (−0.48 to 0.96)	0.51

IOP—intraocular pressure.

**Table 4:** Effect of acetazolamide prophylaxis on BCVA on postoperative day 7.

Variable	Effect Estimate (95% CI)	*p*-Value
Acetazolamide prophylaxis	−0.07 (−0.01 to −0.12)	0.01
Pre-operative BCVA (LogMAR)	0.18 (0.12 to 0.23)	<0.001
Age (years)	0.01 (0 to 0.01)	<0.001
Male	0.05 (0 to 0.1)	0.05

BCVA—Best-Corrected Visual Acuity; LogMAR—logarithm of the Minimum Angle of Resolution.

## Data Availability

The original contributions presented in this study are included in the article. Further inquiries can be directed to the corresponding author.

## References

[1] Lidder A.K., Vanner E.A., Chang T.C., Lum F., Rothman A.L. (2024). Intraocular Pressure Spike Following Stand-Alone Phacoemulsification in the IRIS^®^ Registry (Intelligent Research in Sight). Ophthalmology.

[2] Grzybowski A., Kanclerz P. (2019). Early postoperative intraocular pressure elevation following cataract surgery. Curr. Opin. Ophthalmol..

[3] Slabaugh M.A., Bojikian K.D., Moore D.B., Chen P.P. (2014). Risk factors for acute postoperative intraocular pressure elevation after phacoemulsification in glaucoma patients. J. Cataract Refract. Surg..

[4] Annam K., Chen A.J., Lee I.M., Paul A.A., Rivera J.J., Greenberg P.B. (2018). Risk factors for early intraocular pressure elevation after cataract surgery in a cohort of United States veterans. Mil. Med..

[5] Miller K.M., Oetting T.A., Tweeten J.P., Carter K., Lee B.S., Lin S., Nanji A.A., Shorstein N.H., Musch D.C., American Academy of Ophthalmology Preferred Practice Pattern Cataract/Anterior Segment Panel (2022). Cataract in the adult eye preferred practice pattern. Ophthalmology.

[6] Shingleton B.J., Rosenberg R.B., Teixeira R., O’Donoghue M.W. (2007). Evaluation of intraocular pressure in the immediate postoperative period after phacoemulsification. J. Cataract Refract. Surg..

[7] O’Brien P.D., Ho S.L., Fitzpatrick P., Power W. (2007). Risk factors for a postoperative intraocular pressure spike after phacoemulsification. Can. J. Ophthalmol..

[8] Alwitry A., Rotchford A., Gardner I. (2006). First day review after uncomplicated phacoemulsification: Is it necessary?. Eur. J. Ophthalmol..

[9] Kasetti S.R., Desai S.P., Sivakumar S., Sunderraj P. (2002). Preventing intraocular pressure increase after phacoemulsification and the role of perioperative apraclonidine. J. Cataract Refract. Surg..

[10] Dayanir V., Özcura F., Kir E., Topaloğlu A., Ozkan S.B., Aktunç T. (2005). Medical control of intraocular pressure after phacoemulsification. J. Cataract Refract. Surg..

[11] Rainer G., Menapace R., Findl O., Georgopoulos M., Kiss B., Heinze G. (2000). Randomised fellow eye comparison of the effectiveness of dorzolamide and apraclonidine on intraocular pressure following phacoemulsification cataract surgery. Eye.

[12] Wedrich A., Menapace R. (1992). Intraocular pressure following small-incision cataract surgery and polyHEMA posterior chamber lens implantation. A comparison between acetylcholine and carbachol. J. Cataract Refract. Surg..

[13] Ermis S.S., Ozturk F., Inan U.U. (2005). Comparing the effects of travoprost and brinzolamide on intraocular pressure after phacoemulsification. Eye.

[14] Rainer G., Menapace R., Schmetterer K., Findl O., Georgopoulos M., Vass C. (1999). Effect of dorzolamide and latanoprost on intraocular pressure after small incision cataract surgery. J. Cataract Refract. Surg..

[15] Çetinkaya A., Akman A., Akova Y.A. (2004). Effect of topical brinzolamide 1% and brimonidine 0.2% on intraocular pressure after phacoemulsification. J. Cataract Refract. Surg..

[16] Rainer G., Menapace R., Findl O., Petternel V., Kiss B., Georgopoulos M. (2001). Intraindividual comparison of the effects of a fixed dorzolamide–timolol combination and latanoprost on intraocular pressure after small incision cataract surgery. J. Cataract Refract. Surg..

[17] Bömer T.G., Lagreze W.D., Funk J. (1995). Intraocular pressure rise after phacoemulsification with posterior chamber lens implantation: Effect of prophylactic medication, wound closure, and surgeon’s experience. Br. J. Ophthalmol..

[18] Abbasoglu E., Tekeli O., Celikdogan A., Gürsel E. (2000). A topical or oral carbonic anhydrase inhibitor to control ocular hypertension after cataract surgery. Eur. J. Ophthalmol..

[19] Zohdy G.A., Rogers Z.A., Lukaris A., Sells M., Roberts-Harry T.J. (1998). A comparison of the effectiveness of dorzolamide and acetazolamide in preventing post-operative intraocular pressure rise following phacoemulsification surgery. J. R. Coll. Surg. Edinb..

[20] Beidner B., Rothkoff L., Blumenthal M. (1977). The effect of acetazolamide on early increased intraocular pressure after cataract extraction. Am. J. Ophthalmol..

[21] Zamvar U., Dhillon B. (2005). Postoperative IOP prophylaxis practice following uncomplicated cataract surgery: A UK-wide consultant survey. BMC Ophthalmol..

[22] Holm J.L., Bach-Holm D., Holm L.M., Vestergaard A.H. (2019). Prophylactic treatment of intraocular pressure elevation after uncomplicated cataract surgery in nonglaucomatous eyes—A systematic review. Acta Ophthalmol..

[23] Hayashi K., Yoshida M., Sato T., Manabe S.I. (2019). Effect of topical hypotensive medications for preventing intraocular pressure increase after cataract surgery in eyes with glaucoma. Am. J. Ophthalmol..

[24] Hayashi K., Yoshida M., Manabe S.I., Yoshimura K. (2017). Prophylactic effect of oral acetazolamide against intraocular pressure elevation after cataract surgery in eyes with glaucoma. Ophthalmology.

[25] Hayashi K., Yoshida M., Sato T., Manabe S.I., Yoshimura K. (2018). Intraocular pressure elevation after cataract surgery and its prevention by oral acetazolamide in eyes with pseudoexfoliation syndrome. J. Cataract Refract. Surg..

[26] Blumenthal M., Assia E.I., Chen V., Avni I. (1994). Using an anterior chamber maintainer to control intraocular pressure during phacoemulsification. J. Cataract Refract. Surg..

[27] Chawla H.B., Adams A.D. (1996). Use of the anterior chamber maintainer in anterior segment surgery. J. Cataract Refract. Surg..

[28] Grinbaum A., Blumenthal M., Assia E. (2003). Comparison of intraocular pressure profiles during cataract surgery by phacoemulsification and extracapsular cataract extraction. Ophthalmic Surg. Lasers Imaging.

[29] Malik K.P.S., Goel R. (2009). Nucleus management with Blumenthal technique: Anterior chamber maintainer. Indian J. Ophthalmol..

[30] David R., Zangwill L., Stone D., Yassur Y. (1987). Epidemiology of intraocular pressure in a population screened for glaucoma. Br. J. Ophthalmol..

[31] Rubin D.B. (1997). Estimating causal effects from large data sets using propensity scores. Ann. Intern. Med..

[32] Brízido M., Rodrigues P.F., Almeida A.C., Abegão Pinto L. (2023). Cataract surgery and IOP: A systematic review of randomised controlled trials. Graefes Arch. Clin. Exp. Ophthalmol..

[33] Zetterström C., Behndig A., Kugelberg M., Montan P., Lundström M. (2015). Changes in intraocular pressure after cataract surgery: Analysis of the Swedish National Cataract Register data. J. Cataract Refract. Surg..

[34] Rothman A.L., Chang T.C., Lum F., Vanner E.A. (2023). Intraocular pressure changes following stand-alone phacoemulsification: An IRIS Registry analysis. Am. J. Ophthalmol..

